# Using a Taxonomy to Systematically Identify and Describe Self-Management Interventions Components in Randomized Trials for COPD

**DOI:** 10.3390/ijerph191912685

**Published:** 2022-10-04

**Authors:** Monique Heijmans, Rune Poortvliet, Marieke Van der Gaag, Ana I. González-González, Jessica Beltran Puerta, Carlos Canelo-Aybar, Claudia Valli, Marta Ballester, Claudio Rocha, Montserrat León Garcia, Karla Salas-Gama, Chrysoula Kaloteraki, Marilina Santero, Ena Niño de Guzmán, Cristina Spoiala, Pema Gurung, Saida Moaddine, Fabienne Willemen, Iza Cools, Julia Bleeker, Angelina Kancheva, Julia Ertl, Tajda Laure, Ivana Kancheva, Kevin Pacheco-Barrios, Jessica Zafra-Tanaka, Dimitris Mavridis, Areti Angeliki Veroniki, Stella Zevgiti, Georgios Seitidis, Pablo Alonso-Coello, Oliver Groene, Rosa Sunol, Carola Orrego

**Affiliations:** 1 Netherlands Institute for Health Services Research (NIVEL), 3513 Utrecht, The Netherlands; 2 Avedis Donabedian Research Institute (FAD), 08037 Barcelona, Spain; 3 Faculty of Medicine, Universitat Autònoma de Barcelona (UAB), 08025 Barcelona, Spain; 4 Network for Research on Chronicity, Primary Care, and Health Promotion (RICAPPS), 28029 Madrid, Spain; 5 Iberoamerican Cochrane Centre, Biomedical Research Institute Sant Pau (IIB Sant Pau), 08025 Barcelona, Spain; 6 Department of Primary Education, School of Education, University of Ioannina, 45110 Ioannina, Greece; 7 Knowledge Translation Program, Li Ka Shing Knowledge Institute, St. Michael’s Hospital, Toronto, ON M5B 1W8, Canada; 8 Institute for Health Policy, Management, and Evaluation, University of Toronto, Toronto, ON M5S 3G8, Canada; 9 OptiMedis, 20095 Hamburg, Germany

**Keywords:** COPD, self-management, complex interventions, taxonomy, quality improvement, intervention content

## Abstract

Self-management interventions (SMIs) may improve outcomes in Chronic Obstructive Pulmonary Disease (COPD). However, accurate comparisons of their relative effectiveness are challenging, partly due to a lack of clarity and detail regarding the intervention content being evaluated. This study systematically describes intervention components and characteristics in randomized controlled trials (RCTs) related to COPD self-management using the COMPAR-EU taxonomy as a framework, identifying components that are insufficiently incorporated into the design of the intervention or insufficiently reported. Overall, 235 RCTs published between 2010 and 2018, from a systematic review were coded using the taxonomy, which includes 132 components across four domains: intervention characteristics, expected patient (or caregiver) self-management behaviours, patient relevant outcomes, and target population characteristics. Risk of bias was also assessed. Interventions mainly focused on physical activity (67.4%), and condition-specific behaviours like breathing exercise (63.5%), self-monitoring (50.8%), and medication use (33.9%). Support techniques like education and skills-training, self-monitoring, and goal setting (over 35% of the RCTs) were mostly used for this. Emotional-based techniques, problem-solving, and shared decision-making were less frequently reported (less than 15% of the studies). Numerous SMIs components were insufficiently incorporated into the design of COPD SMIs or insufficiently reported. Characteristics like mode of delivery, intensity, location, and providers involved were often not described. Only 8% of the interventions were tailored to the target population’s characteristics. Outcomes that are considered important by patients were hardly taken into account. There is still a lot to improve in both the design and description of SMIs for COPD. Using a framework such as the COMPAR-EU SMI taxonomy may contribute to better reporting and to better informing of replication efforts. In addition, prospective use of the taxonomy for developing and reporting intervention content would further aid in building a cumulative science of effective SMIs in COPD.

## 1. Introduction

Chronic Obstructive Pulmonary Disease (COPD) is a disease of the airways and lungs that is characterized by a progressive airflow limitation, which is not fully reversible [1,2]. It is one of the major causes of morbidity and mortality worldwide [3]. The economic and social burden related to COPD is expected to increase over the coming decades due to the continued exposure to COPD risk factors such as smoking and air pollution and the increasing aging of the world’s population [4,5].

The aim of COPD treatment is to relieve symptoms, promote quality of life, delay progression, prevent exacerbations, and reduce mortality [1]. Smoking cessation, adequate use of medication, regular physical exercise, and healthy nutrition are the cornerstones of treatment. Whether treatment is successful depends heavily on patients’ self-management [6]. However, the literature suggests that this self-management is often far from optimal [7,8,9]. Many COPD patients find it difficult to cope with the different self-management tasks and need support for this.

Self-management interventions (SMIs) may play a valuable role in supporting people’s self-management, and also in COPD [10,11]. Although different definitions of SMIs exist [12], in general, SMIs can be characterized as supportive interventions that healthcare staff, peers, or laypersons provide to increase patients’ skills and confidence in managing their long-term condition. For COPD, guidelines state that SMIs for people with COPD should be “structured but personalized” [1,10]: they should provide information [11], elicit personalized goals, formulate appropriate strategies, and focus on intrinsic processes (e.g., motivation, resource utilization, coping, and self-efficacy) [1,13] and mental health [14]. Furthermore, behaviour change techniques are recommended to bolster participants’ motivation, confidence, and competence [10].

Recent systematic reviews and meta-analyses of randomized controlled trials (RCTs) of COPD SMIs show positive effects, such as a reduced number of unscheduled physician visits and COPD-related hospital admissions [15], reduced emotional distress, improved health-related quality of life [14], and increased self-efficacy. Two other systematic reviews [16,17] reported less dyspnoea, less healthcare utilization, and improved quality of life in people with COPD who took part in SMIs.

It is important that we learn from effective SMIs and promote their use to patients, professionals, and policymakers to support patients in their self-management and optimized care. SMIs are complex, however, often involving multiple components, being delivered through a range of modalities (i.e., face to face, online, by phone) and across various settings. The complexity of SMIs is further driven by the number and variability of the intervention’s components or support techniques, the target population (e.g., sample size, severity of the disease, etc.), the intensity, and the behavioural change expected in providers and patients. Intervention descriptions should therefore involve more than providing a label or a list of self-management support techniques. Key features—including duration, dose or intensity, mode of delivery, professionals involved, location, targeted outcomes, and behaviours—can all influence effectiveness and replicability. For replication of successful interventions, it is very important that these interventions are described in detail [18]. Unfortunately, this is hardly ever the case. Whilst the body of systematic reviews examining the effectiveness of SMIs is exploding [19], in COPD, few systematic reviews provide sufficient guidance to enable practical application of the synthesized evidence [17,18,20,21]. For example, a study of 137 interventions from 133 RCTs of non-drug interventions found that only 39% of interventions were described adequately in the primary paper or any references, appendices, or websites [22]. Without a complete published description of the intervention, other researchers cannot replicate or build on research findings, and clinicians, patients, and decisionmakers are left unsure about how to reliably implement the intervention in their own environment. 

During the last decades, several classification systems or taxonomies have been developed for describing SMIs in a more systematic manner [11,23,24,25]. Initially, these taxonomies were limited to specific applications, in particular behavioural areas focusing on interventions targeting for example physical activity, alcohol consumption, or smoking cessation [24,26,27]. Later, more comprehensive taxonomies were developed, such as the Behaviour Change Techniques Taxonomy [28]. However, most of these taxonomies focus only on self-management support or behavioural change techniques, with little attention given to other components that are equally relevant to SMI design, implementation, and reporting. 

**Figure 1:**
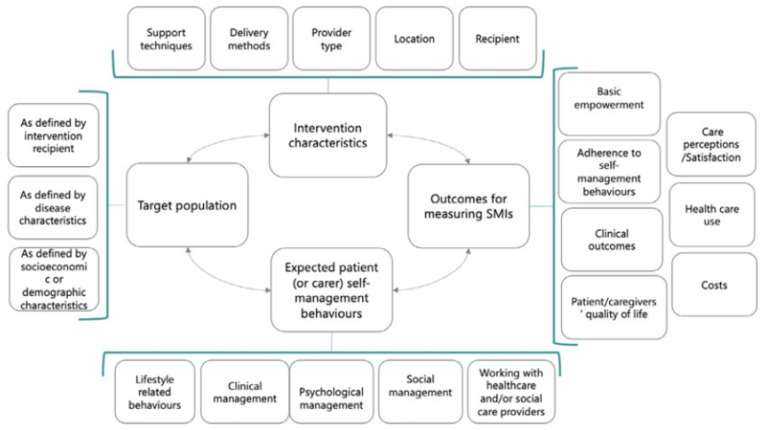
COMPAR-EU self-management intervention taxonomy (Source: Orrego, Health Expect. 2021).

To respond to this shortcoming, we developed a SMI taxonomy within the ”COMPAR-EU Project”. COMPAR-EU is a European Union (EU)-funded project designed to bridge the gap between current knowledge and practice on SMIs that aims to identify the most effective and cost-effective SMIs for four high priority diseases in Europe: diabetes, heart failure, obesity, and COPD [29]. The taxonomy is composed of 132 components, classified in four domains: intervention characteristics, expected patient (or carer) self-management behaviours, type of outcomes to measure self-management interventions, and target population characteristics (Figure 1). It is a generic taxonomy applicable to different chronic diseases. It has been developed through an iterative process based on previous literature and existing taxonomies and was externally validated with a two-round modified online Delphi process, in which 26 international experts on self-management and/or taxonomies participated, including patients ([29], for a description of the developmental process and the final taxonomy). The COMPAR-EU taxonomy differs from other existing taxonomies by its inclusion of additional intervention characteristics that were deemed important for the design, implementation, evaluation, and reporting of SMIs from both a patient’s and professional’s perspective. 

The aim of the present study is to systematically describe intervention components in RCTs related to self-management for COPD using the COMPAR-EU taxonomy as a framework and identify components that are insufficiently incorporated or insufficiently reported into the design of the intervention. Additionally, risk of bias will be assessed. By detecting shortcomings either in the design or in the description of SMIs for COPD, we aim to provide a foundation for the design of new interventions in COPD self-management and contribute to the improvement of the future reporting of interventions. In this way, we facilitate the replication and building on research findings for other researchers and strengthen the available evidence base.

## 2. Materials and Methods

Within COMPAR-EU project, a comprehensive systematic review and network meta-analysis was performed on any SMI for diabetes, obesity, heart failure, and COPD. For this study, only the data for the systematic review for COPD were used. In conducting the work, we followed the rapid reviews method proposed by Cochrane [30].

### 2.1. Information Sources and Literature Search 

We performed a two-stage literature search: first, we drew on the databases of a previous European project (PRO-STEP) (Information available from: https://www.eu-patient.eu/Projects/completed-projects/prostep/ accessed on 5 July 2022), conducted by some of the authors, that identified systematic reviews on SMIs between the years 2000 and 2015 for chronic diseases, including COPD. From this database, we included studies on COPD from 2000 onwards. We updated this search with articles from 2010 up to 5 December 2018 through new searches in MEDLINE, CINAHL, Embase, Cochrane, and PsycINFO. 

The search string for the update was guided by the search string used for PRO-STEP and consisted of at least the key terms ‘self-management’, ‘adults’, ‘COPD’ for content, and ‘randomized controlled trial’ for study type. MESH terms were used to find relevant synonyms for all key terms. Searches were refined by the use of Boolean search operators such as AND, OR, and *. The search algorithms were adapted to the requirements of each database. The full search strategy for each database is attached as an additional file (Appendix A). 

### 2.2. Screening and Selection Process

Our inclusion criteria were 

(i) Population, adults (≥18 years of age) with a diagnosis of COPD, and their caregivers. When a study had a mixed population of patients (i.e., not only people with COPD) and did not report the outcomes per condition separately, it was included if at least 80% of the population targeted COPD; (ii) Intervention, SMIs; (iii) Comparison, usual care (usual care or usual care plus if included self-management support techniques) vs. intervention or an intervention vs. other SMIs (head-to-head); (iv) Outcomes, studies must have reported at least one of the outcomes from the “COMPAR-EU” predefined COS [31]. (v) Study design, RCTs; quasi-randomized studies were excluded. We included only studies published in English or Spanish. More details about the inclusion and exclusion criteria can be found in Appendix B. 

### 2.3. Data Extraction and Synthesis

For data extraction, a pre-designed extraction platform was used. This data extraction platform followed the COMPAR-EU taxonomy structure of SMIs (Figure 1 and included expected patient (or caregiver) self-management behaviours, intervention characteristics, outcomes, target population characteristics (guided by the taxonomy), and information on study design and risk of bias (see also Appendix C). 

Expected patient (or caregiver) self-management behaviours:

A distinction is made between lifestyle-related behaviours, clinical management, psychological management, social management, and working with healthcare. 

Intervention characteristics:

Intervention characteristics include information on self-management support techniques, mode of delivery, providers involved, location, and the recipients of the intervention.

Outcomes:

For the outcomes, a core outcomes set of 15 outcomes was used (see Appendix D) that was developed together with COPD patients and professionals in an earlier stage of the COMPAR-EU project [32]. This COPD-specific outcome set covers almost all the main outcome categories of the generic COMPAR-EU taxonomy (Figure 1).

Target population characteristics:

A distinction was made between disease-related characteristics (time since diagnosis, disease severity and comorbidities) and socio-economic or demographic characteristics (age, sex, socio-economic status, cultural group, and (digital-) health literacy).

Risk of bias

One author assessed each study for risk of bias using Cochrane’s tool for assessing risk of bias in included studies, and then a second reviewer verified the judgments [33]. When there was not enough information reported to decide, we contacted the study authors and requested clarification or further information. We rated the risk of bias as low risk, high risk, or unclear for all included studies in each of the five domains of the Cochrane ‘Risk of bias’ tool [32].

The data extraction platform provides detailed definitions of each of the variables to be extracted and coding rules and examples. During the extraction phase, we added coding to some of the variables based on discussions between coders during structural discrepancy-resolution meetings. Data extraction for COPD was done in the period from January 2020 until May 2020.

A handbook was developed following Cochrane guidance, and training sessions were held for those responsible for collecting data. Before the extraction process, all reviewers (*n* = 10) were trained and underwent calibration to ensure interrater agreement (>80%). After a calibration exercise, two reviewers independently screened the search results to select potentially eligible records based on title and abstract. Subsequently, two reviewers independently confirmed eligibility based on the full text articles of the relevant selected records. In case of disagreement, they reached consensus by discussion or involving a third reviewer. We used Covidence© (www.covidence.org, accessed on 3 February 2018) for the article’s selection. Through the whole data extraction process, a team of experienced supervisors, all experts on self-management and chronic disease, were available to the reviewers for guidance.

Additionally, all collected data were reviewed by an independent researcher (peer reviewer) to ensure quality. For some SMIs, there were multiple publications. These were combined in one extraction. If the paper(s) of a selected study contained too little information to fill in the extraction form, the original protocol paper of that study was also searched to make the extraction as complete as possible.

### 2.4. Data Analysis

We quantified the frequency of identified taxonomy components across intervention arms of selected studies on SMIs for COPD. The results will be organized within the four domains of the taxonomy: intervention characteristics, expected patient (or caregiver) self-management behaviours, type of outcomes to measure self-management interventions, target population characteristics, and on risk-of bias information. Therefore, we focused on the components that would teach us about the design and reporting of the intervention and not on the results or effectivity of the interventions. Results on effectiveness will be published elsewhere.

## 3. Results

### 3.1. Included Studies

**Figure 2:**
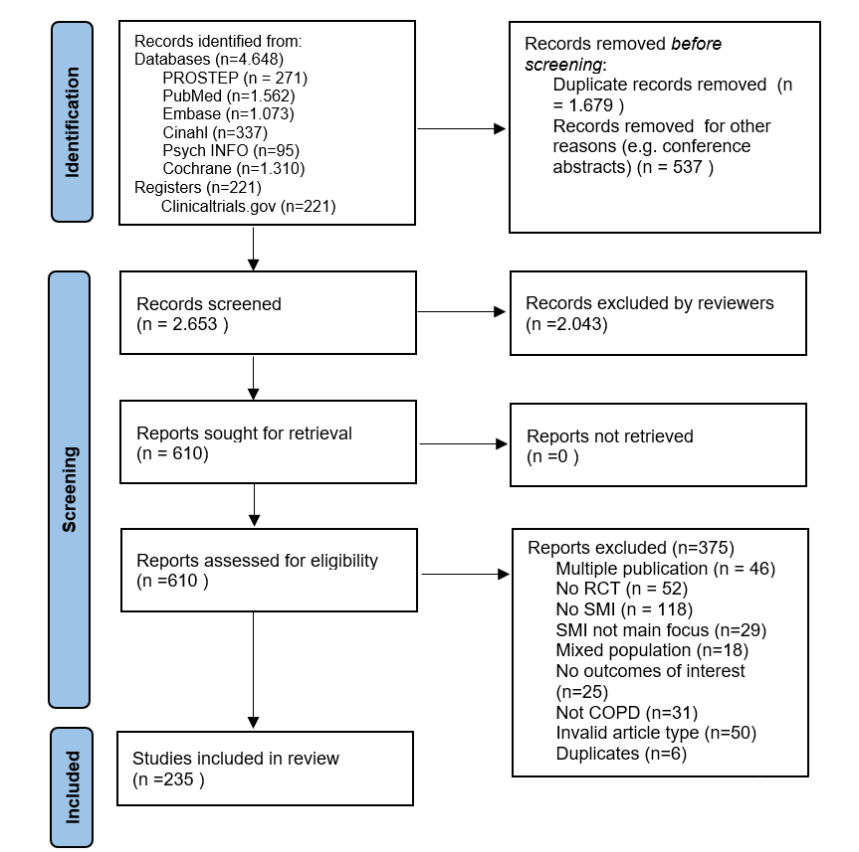
Preferred Reporting Items for Systematic Reviews and Meta-Analyses (PRISMA) flow chart [33].

A total of 4.869 studies were initially identified. From these studies, 2.216 were removed before screening because of being duplicates or conference abstracts. After title and abstract screening of the remaining 2.653 studies by two reviewers, 610 studies remained for full-text screening. Finally, 235 studies were included. The main reasons for exclusion (*n* = 375) during the extraction phase were being no SMI, no RCT, a multiple publication, or an invalid publication type (Figure 2).

### 3.2. Key Characteristics of Included Studies

Of the 235 included studies, 184 (78%) compared one or more SMIs to usual care, and 51 (22%) compared two or more intervention arms (range 2–4), making a total of 184 usual care arms and 307 intervention arms. In 97% of the studies, the intervention focused only on patients; in 4 studies (2%) family or caregivers were involved; 63% of the studies were single centre studies, 32% multi-centre studies, and in 5% this was unclear or not reported.

The studies were conducted in 32 countries: 45% in Europe (mainly the UK and the Netherlands) and 55% outside Europe (mainly the Unites States, China, and Australia). Studies were only implemented in the country of origin and not implemented elsewhere. Although 80% of the studies were published after 2010, interventions were conducted between 1994 and 2016, with 37% starting in the last decade; 8% of the interventions reported explicitly that the intervention was tailored or personalised to the study population.

### 3.3. Usual Care Arms

Information about how usual care was organized in the included trials was limited: 58% of the studies including usual care (*n* = 184) did not provide information on self-management support techniques used in usual care; 60% of the usual care descriptions did not specify targeted self-management behaviours; and in 66–72% of the usual care arms (depending on the component), information about delivery methods, location, providers, type and intensity of support methods was missing. This makes it difficult to compare usual care and intervention arms in detail. However, in studies with a usual care arm that provided information on what constituted usual care, it seemed to consist of some form of information or education on smoking reduction, medication use, eating or physical activity. Most of the time, this information is given during outpatient clinical visits to individual patients, by a nurse or a physician.

### 3.4. Intervention Arms

#### 3.4.1. Expected Patient or Carer Self-Management Behaviours

In the intervention arms (*n* = 307), the number of expected behaviours varied between 0 and 12 (median 3, IQR 2–5). Expected behaviours most often mentioned were lifestyle-related behaviours, like being physically active (67%), and those related to clinical management, especially condition-specific behaviour like breathing exercises (64%), self-monitoring (51%), and medication use (34%); behaviours in relation to psychological or social management and the interaction with healthcare were far less mentioned (Table 1). Combinations of condition-specific behaviours with physical activity, medication use, and/or self-monitoring were often observed.

#### 3.4.2. Reported Intervention Characteristics

- Self-management support techniques

All intervention descriptions (*n* = 307) reported on self-management support techniques, although the number of support techniques in the studies varied between 1 and 10 (Median 4, IQR 3–5). The support techniques most frequently reported were sharing information (95%), skills training (69%), and self-monitoring (47%). Goal setting and provision of equipment were used in one third of the studies. Emotional management was mentioned by 19% of the intervention arms. Shared decision-making was explicitly mentioned in only 2% of the interventions (Table 2). Combinations of sharing information and skills training, sharing information and self-monitoring, and equipment provision in combination with sharing information and/or skills training were also reported.

- Mode of delivery and setting

Almost all studies provided information about mode of delivery, setting, and professionals involved, although the amount of detail differed widely (Table 3): 56% of the COPD interventions used support sessions, and 24% used a combination of these sessions with clinical visits and self-guidance. Half of the intervention arms used face-to-face contact, and 36% used a combination of face-to-face and remote contacts. Most interventions took place in a single location (62%) and 33% in multiple locations, most often two (not in table). Outpatient care (57%) and homecare (45%) were most often chosen as locations, and were delivered by a nurse, physiotherapist, or physician in most cases. SMIs for COPD were given less often in hospitals or in a virtual setting, and almost never in long-term care facilities, in a community setting, or at work. Information on location was missing in 5% of the studies, and information on type of provider in 25% of the studies.

- Duration, intensity, and follow-up

The reporting of duration or intensity was poor and widely variable. For face-to face as well as remote contacts, around 50% had no information on both duration and intensity (not in table).

#### 3.4.3. Outcomes

There was a lot of heterogeneity in the frequency with which outcomes from the COS appeared in the study (Table 4). Quality of life (66%), physical activity/muscle strength (56%), frequency of short-term COPD symptoms like dyspnoea, cough, and sputum (43%), emergency room visits (36%), and the ability to cope with the disease (23%) were reported most often. Outcomes related to basic empowerment strategies such as self-efficacy knowledge and patient activation were reported less often. The way outcomes were measured either by questionnaire, observation, or medical examination across studies also differed a lot, as well as the measurement tools (Table 4).

#### 3.4.4. Target Population Characteristics

Target populations were mainly described by sex, age, and illness severity, using FEV1 (85%) and the GOLD Classification (10%). Only 17% of the studies reported on illness duration, and 14% on comorbidity, depression being the most common comorbidity (16%). Less than 2% of the studies focused specifically on populations with lower SES or from minority groups, and 8% of the intervention arms were tailored to the target population. In about half of the studies, a threshold for age and/or illness severity was used as an inclusion criterion. Other variables almost never played a role as inclusion criteria (Table 5).

#### 3.4.5. Risk of Bias in Included Studies

**Figure 3:**
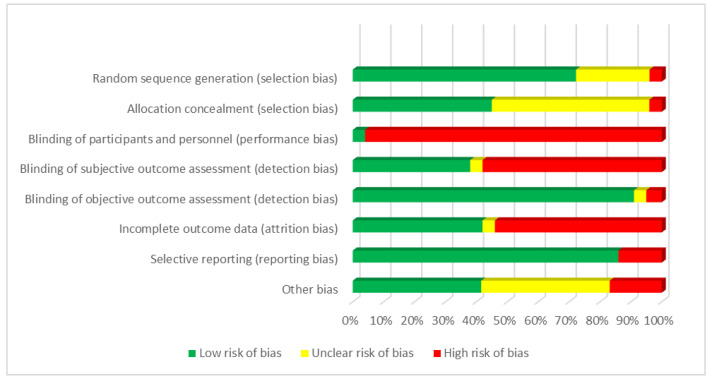
Risk of bias of included COPD SMIs.

A summary of our judgment for each potential risk of bias across the included studies is provided in Figure 3. Most studies had a low risk of bias in the sequence generation of the random number for allocation of participants, but methods for concealment of the allocation were poorly reported and thus an important proportion was judged as unclear. The main methodological limitation of the included studies was the lack of blinding of participants and personnel, as very few studies incorporated a procedure to hide the active group from other participants or care personnel or used a “sham” intervention to reduce the influence of being aware of which arm participants were allocated to. This limitation of lack of blinding of outcome assessment also affected the assessment of the subjective outcomes (quality of life, depression, anxiety, dyspnoea symptoms, self-efficacy, knowledge, etc.), and objective outcomes that might be influenced by the assessor (i.e., FEV1, FEV/FVC, etc.). We considered that objective outcomes assessments from laboratory tests or based in clearly observed events (i.e., mortality, hospitalization, and exacerbations) were not affected by the lack of blinding.

Around 50% of the studies also had a relevant number of drop-out during follow-up, raising concerns due to attrition (Figure 2). The risk of selective reporting was more difficult to evaluate, as few studies made their protocols available before the publication of the results.

## 4. Discussion

### 4.1. Summary of Evidence

The aim of this study was to systematically describe the intervention components and design of RCTs on COPD self-management by using the recently developed COMPAR-EU SMI Taxonomy.

The descriptive analysis shows that interventions for COPD use a number of support techniques, most frequently using sharing information, skills training in the correct use of medication, and self-monitoring of symptoms. Support techniques to improve motivation for self-management or self-efficacy were reported less often, however. Additionally, goal setting was used in less than one third of the studies. With regard to the outcomes used to measure the effects, only three of the 15 outcomes that are considered very important by patients and professionals [31] were regularly included in studies: quality of life, physical activity, and short-term COPD symptoms were measured in over 40% of the studies. Other outcome measures, such as self-efficacy, mortality, exacerbation rate, smoking cessation, and patient activation, were measured in less than 10% of the studies.

Regarding the design of SMIs for COPD, the results of this study show that in only 8% of the interventions was it explicitly stated that the information was tailored, which means that the content of the intervention or the way it was delivered was tailored or personalized to the study population. For example, educational material was simplified because of COPD patients with low health literacy, translated because of Spanish speaking patients, or adapted because of known gender differences between men and women. Looking at the reporting of other design characteristics like mode of delivery, intensity, location, and involved providers, there was suboptimal reporting on these variables and a huge variety in the way these variables were reported across studies. The same counts for how outcomes in studies were operationalized and measured. Besides that, this complicates the replication and implementation of successful interventions, and also makes it difficult to compare and evaluate interventions. Another complicating factor was that usual care was often poorly described with almost no information on self-management support techniques, targeted behaviours, delivery modes, location, providers, type, and intensity of support. This makes it difficult to compare usual care and intervention arms and determine the (cost-)effectiveness of interventions compared to usual care. Additionally, with respect to the quality of the studies, it was concluded that reaching high quality according to the quality rules of RCTs is difficult for this type of study in the area of SMIs. Although in most studies we found a high or unclear risk of bias in more than three of the domains assessed (performance bias, detection bias, attrition bias), in the case of SMIs blinding, for example, is difficult, which especially may influence the assessment of subjective outcomes that are often used in SMIs. One may wonder whether SMIs need their own quality rules. In summary, the results make perfectly clear that there is still a lot to improve in both the design and description of COPD SMI interventions. Using a taxonomy for the description of intervention and design components turned out to be useful, as we were able to identify the weaknesses in the design and reporting of interventions in COPD SMIs. Taxonomies such as the COMPAR-EU SMI taxonomy provide a common language and definitions for understanding the content of SMIs across contexts. Although the COMPAR-EU taxonomy is a generic taxonomy applicable to different chronic diseases, it can also very well be applied disease-specifically by focusing more on ingredients of SMIs that matter for a specific disease in certain parts of the taxonomy. This applies, in particular, to the components of targeted behaviours, support techniques, and outcomes, which can differ for each disease.

### 4.2. Limitations and Strengths

The strengths of this study are a very large set of RCTs, a rigorous methodology, and the use of a taxonomy and set of outcomes that was developed together with patients, professionals, and implementation experts, guaranteeing the relevance of our findings for practice. A limitation of this study was that the selected interventions included the period until 2018. Therefore, it is possible that some of the areas with limited information reported have improved in recent years, though in some cases there were so few studies that it is likely that the detected gaps will still be valid. This is confirmed in a number of recent systematic reviews of self-management interventions in COPD that included studies of more recent date. They also stress the lack of patient relevant outcomes in studies evaluating SMIs in COPD [34], the heterogeneity in design, and the lack of sufficient detail in intervention descriptions which complicates the evaluation of these interventions and the use of these interventions in clinical practice [34,35,36]. However, an update of this study in a few years would be valuable given the speed at which SMIs are developing.

### 4.3. Our results in the Context of Previous Research

Although opinions in literature differ about which components should be included into the design of COPD SMIs, there is general agreement that they should at least be “personalized … with goals of motivating, engaging and supporting the participants to positively adapt their behaviour(s) and develop skills to better manage their disease” [1]. In this context, our finding that only 8% of the RCTs were tailored is quite low, given that between 30% and 60% of the COPD population has low health literacy worldwide [37,38], the prevalence of COPD differs among ethnic groups [39], and that men and women differ in response to available therapeutic modalities [40]. There is also agreement that SMIs for COPD should focus on intrinsic processes (e.g., motivation, resource utilization, coping, and self-efficacy) [1,10,13] and mental health [14]. All these aspects were addressed in less than 20% of the studies. Therefore, key competences that COPD patients need in order to play an active role in their own treatment are less supported. From our own research, we know that patients themselves often like to play an active role in their own treatment but are less supported by professionals in this. Prejudices that COPD patients are too old, too sick, or less motivated seem to play a role here [35]. Patients rated self-efficacy, coping with the disease, and patient activation as one of the most important outcomes of SMIs [32]. This stresses the importance of exploring mutual expectations and roles during the self-management support and to make agreements about this. Additionally, emotional management was only mentioned by 19% of the interventions, whereas this is seen as an important part of COPD treatment [10], and anxiety and depression are important emotional comorbidities of COPD [41] that may hinder self-management. Last but not least, it is important that interventions focus on the improvement of those outcomes that that are important for patients. Not only will this increase adherence, but it will also contribute to more personalized healthcare and more informed health decisions in clinical practice [32]. Our finding that only three out of fifteen core outcomes were addressed in over 40% of the studies is quite disappointing. This questions the degree of person-centeredness of the studies found. It is important to determine treatment choices and treatment goals in consultation with patients. Increasingly, and with success, patients are involved in the design of interventions (co-creation), which may highly improve the quality of and adherence to these interventions [42].

### 4.4. Implications for Practice and Research

Including intervention descriptions for COPD SMI components and design characteristics in the areas with less information detected could help to get a better picture on the reported results of SMIs, their value, and how they can be replicated. Shortcomings in intervention reporting and design are not specific to self-management research [43]. However, their detrimental effect on the building of a solid evidence base is likely amplified by the inherent complexity of the interventions delivered in these areas of work.

During the screening phase, we encountered many RCTs for COPD containing supervised exercises. These studies were excluded as they exceed support levels coherent with the definition of SMIs. The same was true for RCTs describing interventions that were part of a disease-management program or COPD rehabilitation. They were excluded if the effects of the SMI could not be separated from the disease-management program as a whole. Describing interventions in a more uniform and systematic way and connecting with what is important from the perspective of COPD patients are important steps. Additionally, more agreement about what constitutes self-management and what does not is of utmost relevance. Given the pressing need for effective SMI in chronic disease, these steps may, if addressed, strengthen the available evidence base and contribute to better and more person-centred healthcare.

## 5. Conclusions

Many RCTs in COPD to promote self-management are poorly described or do not adequately fit the needs and possibilities of patients. Poor intervention description hinders the replication and building on research findings for other researchers. A lack of tailoring contributes to suboptimal healthcare. The COMPAR-EU SMI Taxonomy is a useful tool for characterising SMI content in more detail and offers a promising way forward in identifying and analysing the components and characteristics of interventions. Prospective use of taxonomies for developing and reporting content would further aid in building a cumulative evidence base.

## Figures and Tables

**Table 1:** Expected self-management behaviours from COPD SMI-descriptions.

	Intervention Arms
	*n* = 307
Median number of expected behaviours	3.0 (2.0–5.0)
**Lifestyle-related behaviours**	
Physical activity	207 (67.4%)
Healthy eating	85 (27.7%)
Smoking reduction	69 (22.5%)
Healthy sleep	11 (3.6%)
Alcohol reduction	5 (1.6%)
**Clinical management**	
Condition-specific behaviour (e.g., breathing exercise)	195 (63.5%)
Self-monitoring	156 (50.8%)
Medication use	104 (33.9%)
Early recognition of symptoms	71 (23.1%)
Managing devices (e.g., inhaler, oxygen)	53 (17.3%)
Physical limitations management	27 (8.8%)
**Psychological management**	
Handling emotions	76 (24.8%)
**Social management**	
Combining COPD with social roles	7 (2.3%)
Being fit enough for work	2 (0.7%)
Being able to work	1 (0.3%)
**Working with healthcare/providers**	
Communication with healthcare (providers)	23 (7.5%)
Asking for professional help	35 (11.4%)

**Table 2:** Self-management support techniques from intervention descriptions (*n* = 235).

	Intervention Arms
	*n* = 307
**Median number of support techniques**	4.0 (3.0–5.0)
**Type of support technique**	
Sharing information	290 (94.5%)
Skills training	212 (69.1%)
Self-monitoring	144 (46.9%)
Goal setting	110 (35.8%)
Equipment provision	97 (31.6%)
Emotional management	58 (18.9%)
Coaching	55 (17.9%)
Enhancing problem solving	40 (13.0%)
Social support	33 (10.7%)
Prompts use	32 (10.4%)
Services use	30 (9.8%)
Shared decision-making	7 (2.3%)
No specific self-management	0 (0.0%)

Data are presented as median (IQR) for continuous measures, and *n* (%) for categorical measures.

**Table 3:** Mode of delivery, setting, and providers involved from intervention descriptions (*n* = 307).

	*n* (%)		*n* (%)
**Mode of delivery**		**Time of communication**	
Clinical visits	30 (9.8%)	Synchronous	231 (75.2%)
Support sessions	172 (56.0%)	A-synchronous	18 (5.9%)
Self-guided	29 (9.4%)	Combination	58 (18.9%)
Combination	75 (24.4%)	Not reported	0 (0.0%)
Not reported	1 (0.0%)		
	***n*** **(%)**		***n*** **(%)**
**Type of contact**		**Population**	
Face-to-Face	152 (49.5%)	Groups	83 (26.0%)
Remote	42 (13.7%)	Individual	224 (71.0%)
Combination	109 (35.5%)	Combination	9 (3%)
Not reported	4 (1.3%)	Not reported	0 (0%)
	***n*** **(%)**		***n*** **(%)**
**Location (Top 5)**		**Provider (Top 5)**	
Outpatient care	176 (57.3%)	Nurse	117 (38.1%)
Homecare	138 (45.0%)	Physiotherapist	84 (27.4%)
Hospital care	31 (10.1%)	Physician	53 (17.3%)
Virtual	29 (9.4%)	Online service	33(10.7%)
Community care	12 (3.9%)	Educator	26 (8.5%)

**Table 4:** Frequency of outcomes used in SMI for COPD *.

	Total
	*n* = 235 *n* (%)
**Basic empowerment**	
Self-efficacy	31 (13.2%)
Knowledge	12 (5.1%)
Patient activation	4 (1.7%)
**Adherence to SM-behaviours**	
Physical activity/Muscle strength	131 (55.7%)
Adherence to treatment and recommended visits	13 (5.5%)
Smoking Cessation	11 (4.7%)
**Clinical outcomes**	
COPD symptoms (short-term)	101 (43.0%)
Lung Function	56 (23.8%)
Mortality	24 (10.2%)
Exacerbation	22 (9.4%)
**Patients/caregivers quality of life**	
Quality of life	154 (65.5%)
Coping with the disease, including depression and anxiety	55 (23.4%)
Activities of daily living	14 (6.0%)
Sleep Quality	3 (1.3%)
**Healthcare use**	
Number of emergency room visits and admissions	85 (36.2%)

* Outcomes from Core Outcome Set defined by COPD patients and professionals.

**Table 5:** Reported population characteristics from study descriptive (*n* = 235).

Characteristic	Reported (*n*/%)	Used as Inclusion Criteria (*n*/%)
Sex	222 (94.5%)	4 (1.7%)
Age	230 (97.9%)	121 (51.1%)
Socio-economic status *	4 (1.7%)	0 (0.0%)
Cultural background *	3 (1.3%)	2 (0.9%)
Health Literacy level	0 (0.0%)	0 (0.0%)
Time since diagnosis	39 (16.6%)	2 (0.9%)
Comorbidities	32 (13.6%)	7 (3.0%)
Illness severity	214 (91.1%)	135 (57.4%)

* Only extracted as reported when over 80% of the study population had a low socio-economic status or specific cultural background.

## Data Availability

Data is contained within the article; database of original papers from review can be provided by contacting the first author.

## Appendix A

Literature searches performed on 5 December 2018 for all databases

**Search History PubMed 8 December 2018**

Search	Query	Items found
#3	#1 AND #2 AND #3 Filters: Publication date from 2010/01/01	1562
#4	#1 AND #2 AND #3	2787
#3	(“Clinical Trials as Topic”[Mesh] OR “Randomized Controlled Trial” [pt] OR “Controlled Clinical Trial” [pt] OR randomized [tiab] OR randomly[tiab] OR placebo[tiab] OR trial[ti]) NOT (animals[mh] NOT humans[mh])	1,167,356
#2	“Self Care”[Mesh] OR “Self-Management”[Mesh] OR “Power (Psychology)”[Mesh] OR “Health Education”[Mesh] OR “Patient Participation”[Mesh] OR “Decision Making”[Mesh] OR “Telemedicine”[Mesh] OR self-administration*[tiab] OR self-care[tiab] OR self efficac*[tiab] OR self-manag*[tiab] OR selfmanag*[tiab] OR self-monitor*[tiab] OR selfmonitor*[tiab] OR self-diagnos*[tiab] OR selfdiagnos*[tiab] OR self-assess*[tiab] OR selfassess*[tiab] OR self-direct*[tiab] OR selfdirect*[tiab] OR self-help*[tiab] OR empower*[tiab] OR enablement[tiab] OR health education[tiab] OR patient education[tiab] OR patient participation[tiab] OR coach*[tiab] OR health promot*[tiab] OR ((community[tiab] OR peer[tiab]) AND (support*[tiab] OR advice*[tiab] OR monitor*[tiab] OR train*[tiab] OR instruction*[tiab] OR intervention*[tiab] OR consult*[tiab] OR assist*[tiab] OR educat*[tiab] OR information*[tiab] OR skill*[tiab])) OR group support*[tiab] OR group intervention*[tiab] OR group advice*[tiab] OR group monitor*[tiab] OR group train*[tiab] OR training group*[tiab] OR group instruct*[tiab] OR group assist*[tiab] OR group educat*[tiab] OR group inform*[tiab] OR group skill*[tiab] OR ((patient[tiab] OR patients[tiab]) AND (centered[tiab] OR centred[tiab] OR focus*[tiab] of directed[tiab] OR coach*[tiab] OR engage*[tiab] OR involve*[tiab] OR orient*[tiab] OR participat*[tiab])) OR ((patient[tiab] OR patients[tiab]) AND (educat*[tiab] OR train*[tiab] OR instruct*[tiab] OR teach*[tiab])) OR (management*[tiab] AND (plan[tiab] OR program*[tiab] OR disease[tiab])) OR (symptom*[tiab] AND (management[tiab] OR directed[tiab] OR focus*[tiab])) OR ((personalized[tiab] OR personalized[tiab]) AND care[tiab]) OR telemedicine[tiab] OR eHealth[tiab] OR e-Health[tiab] OR mHealth[tiab] OR m-Health[tiab] OR shared decision*[tiab] OR sharing decision*[tiab] OR informed decision*[tiab] OR informed choice*[tiab] or decision aid*[tiab] OR ((share*[ti] OR sharing*[ti] OR informed*[ti]) AND (decision*[ti] OR deciding*[ti] OR choice*[ti]))	1,904,809
#1	“Pulmonary Disease, Chronic Obstructive”[Mesh] OR COPD[tiab] OR obstructive lung disease*[tiab] OR obstructive pulmonary disease*[tiab] OR acute exacerbation*[tiab] OR AECB[tiab] OR restrictive lung disease*[tiab] OR chronic pulmonary obstructive disease*[tiab] OR COAD[tiab] OR chronic obstructive airway disease*[tiab]	85,951

**Search History Embase 8 December 2018**

Search	Query	Items found
#6	#5 NOT (‘chapter’/it OR ‘conference abstract’/it OR ‘conference review’/it OR ‘editorial’/it OR ‘letter’/it OR ‘note’/it OR ‘short survey’/it)	1073
#5	#1 AND #2 AND #3 AND [2010–2019]/py	1494
#4	#1 AND #2 AND #3	1995
#3	(‘clinical trial (topic)’/exp OR randomized:ab,ti,kw OR randomly:ab,ti,kw OR placebo:ab,ti,kw OR trial:ti) NOT ([animals]/lim NOT [humans]/lim)	1,267,704
#2	‘self care’/exp OR ‘coping behaviour’/exp OR ‘drug self administration’/exp OR ‘self monitoring’/exp OR ‘empowerment’/exp OR ‘health education’/exp OR ‘patient participation’/exp OR ‘shared decision making’/exp OR ‘telehealth’/exp OR ‘self administration*’:ab,ti,kw OR ‘self care’:ab,ti,kw OR ‘self efficac*’:ab,ti,kw OR ‘self manag*’:ab,ti,kw OR selfmanag*:ab,ti,kw OR ‘self monitor*’:ab,ti,kw OR selfmonitor*:ab,ti,kw OR ‘self diagnos*’:ab,ti,kw OR selfdiagnos*:ab,ti,kw OR ‘self assess*’:ab,ti,kw OR selfassess*:ab,ti,kw OR ‘self direct*’:ab,ti,kw OR selfdirect*:ab,ti,kw OR ‘self help*’:ab,ti,kw OR empower*:ab,ti,kw OR enablement:ab,ti,kw OR ‘health education’:ab,ti,kw OR ‘patient education’:ab,ti,kw OR ‘patient participation’:ab,ti,kw OR coach*:ab,ti,kw OR ‘health promot*’:ab,ti,kw OR (((group OR peer) NEAR/3 (support* OR advice* OR monitor* OR intervention* OR train* OR instruction* OR consult* OR assist* OR educat* OR information* OR skill*)):ab,ti,kw) OR (((patient OR patients) NEAR/3 (centered OR centred OR focus* OR directed OR coach* OR engage* OR involve* OR orient* OR participat*)):ab,ti,kw) OR (((patient OR patients) NEAR/3 (educat* OR train* OR instruct* OR teach*)):ab,ti,kw) OR ((management* NEAR/3 (plan OR program* OR disease)):ab,ti,kw) OR ((symptom* NEAR/3 (management OR directed OR focus*)):ab,ti,kw) OR (((personalized OR personalized) NEAR/3 care):ab,ti,kw) OR telemedicine:ab,ti,kw OR ehealth:ab,ti,kw OR ‘e health’:ab,ti,kw OR mhealth:ab,ti,kw OR ‘m health’:ab,ti,kw OR ‘shared decision*’:ab,ti,kw OR ‘sharing decision*’:ab,ti,kw OR ‘informed decision*’:ab,ti,kw OR ‘informed choice*’:ab,ti,kw OR ‘decision aid*’:ab,ti,kw OR (((share* OR sharing* OR informed*) NEAR/3 (decision* OR deciding* OR choice*)):ab,ti,kw)	972,108
#1	‘chronic obstructive lung disease’/exp OR (obstructive NEAR/3 (lung OR pulmonary OR airway) NEAR/3 disease*):ab,ti,kw OR ‘acute exacerbation*’:ab,ti,kw OR AECB:ab,ti,kw OR ‘restrictive lung disease*’:ab,ti,kw OR COAD:ab,ti,kw	136,078

**Search History psycINFO 8 December 2018**

Search	Query	Items found
S5	S1 AND S2 AND S3 Limiters—Publication Year: 2010–2018	95
S4	S1 AND S2 AND S3	153
S3	DE “Clinical Trials” OR TI (randomized OR randomly OR placebo OR trial) OR AB (randomized OR randomly OR placebo) OR KW (randomized OR randomly OR placebo OR trial) NOT (PO Animal NOT PO Human)	165,282
S2	DE (“Self-Care Skills” OR “Self-Efficacy” OR “Drug Self Administration” OR “Coping Behavior” OR “Empowerment” OR “Telemedicine” OR “Health Education” OR “Health Promotion” OR “Client Participation”) OR TI (“self administration*” OR “self care” OR “self efficac*” OR “self manag*” OR selfmanag* OR “self monitor*” OR selfmonitor* OR “self diagnos*” OR selfdiagnos* OR “self assess*” OR selfassess* OR “self direct*” OR selfdirect* OR “self help*” OR empower* OR enablement OR “health education” OR “patient education” OR “patient participation” OR coach* OR “health promot*” OR (((group OR peer) N3 (support* OR advice* OR monitor* OR intervention* OR train* OR instruction* OR consult* OR assist* OR educat* OR information* OR skill*))) OR (((patient OR patients) N3 (centered OR centred OR focus* OR directed OR coach* OR engage* OR involve* OR orient* OR participat*))) OR (((patient OR patients) N3 (educat* OR train* OR instruct* OR teach*))) OR ((management* N3 (plan OR program* OR disease))) OR ((symptom* N3 (management OR directed OR focus*))) OR (((personalized OR personalized) N3 care)) OR telemedicine OR ehealth OR “e health” OR mhealth OR “m health” OR “shared decision*” OR “sharing decision*” OR “informed decision*” OR “informed choice*” OR “decision aid*” OR (((share* OR sharing* OR informed*) N3 (decision* OR deciding* OR choice*))) OR AB (“self administration*” OR “self care” OR “self efficac*” OR “self manag*” OR selfmanag* OR “self monitor*” OR selfmonitor* OR “self diagnos*” OR selfdiagnos* OR “self assess*” OR selfassess* OR “self direct*” OR selfdirect* OR “self help*” OR empower* OR enablement OR “health education” OR “patient education” OR “patient participation” OR coach* OR “health promot*” OR (((group OR peer) N3 (support* OR advice* OR monitor* OR intervention* OR train* OR instruction* OR consult* OR assist* OR educat* OR information* OR skill*))) OR (((patient OR patients) N3 (centered OR centred OR focus* OR directed OR coach* OR engage* OR involve* OR orient* OR participat*))) OR (((patient OR patients) N3 (educat* OR train* OR instruct* OR teach*))) OR ((management* N3 (plan OR program* OR disease))) OR ((symptom* N3 (management OR directed OR focus*))) OR (((personalized OR personalized) N3 care)) OR telemedicine OR ehealth OR “e health” OR mhealth OR “m health” OR “shared decision*” OR “sharing decision*” OR “informed decision*” OR “informed choice*” OR “decision aid*” OR (((share* OR sharing* OR informed*) N3 (decision* OR deciding* OR choice*))) OR KW (“self administration*” OR “self care” OR “self efficac*” OR “self manag*” OR selfmanag* OR “self monitor*” OR selfmonitor* OR “self diagnos*” OR selfdiagnos* OR “self assess*” OR selfassess* OR “self direct*” OR selfdirect* OR “self help*” OR empower* OR enablement OR “health education” OR “patient education” OR “patient participation” OR coach* OR “health promot*” OR (((group OR peer) N3 (support* OR advice* OR monitor* OR intervention* OR train* OR instruction* OR consult* OR assist* OR educat* OR information* OR skill*))) OR (((patient OR patients) N3 (centered OR centred OR focus* OR directed OR coach* OR engage* OR involve* OR orient* OR participat*))) OR (((patient OR patients) N3 (educat* OR train* OR instruct* OR teach*))) OR ((management* N3 (plan OR program* OR disease))) OR ((symptom* N3 (management OR directed OR focus*))) OR (((personalized OR personalized) N3 care)) OR telemedicine OR ehealth OR “e health” OR mhealth OR “m health” OR “shared decision*” OR “sharing decision*” OR “informed decision*” OR “informed choice*” OR “decision aid*” OR (((share* OR sharing* OR informed*) N3 (decision* OR deciding* OR choice*)))	410,566
S1	DE “Chronic Obstructive Pulmonary Disease” OR TI (obstructive N3 (lung OR pulmonary OR airway) N3 disease*) OR “acute exacerbation*” OR AECB OR “restrictive lung disease*” OR COAD) OR AB (obstructive N3 (lung OR pulmonary OR airway) N3 disease*) OR “acute exacerbation*” OR AECB OR “restrictive lung disease*” OR COAD) OR KW (obstructive N3 (lung OR pulmonary OR airway) N3 disease*) OR “acute exacerbation*” OR AECB OR “restrictive lung disease*” OR COAD)	2794

**Search History Cinahl 8 December 2018**

Search	Query	Items found
S5	S1 AND S2 AND S3 Limiters—Publication Year: 2010–2018, academic journals	337
S4	S1 AND S2 AND S3	668
S3	MH “Clinical Trials+” OR TI (randomized OR randomly OR placebo OR trial) OR AB (randomized OR randomly OR placebo) NOT (MH “Animals” NOT MH “Human)	377,931
S2	MH (“Self Care+” OR “Self-Efficacy” OR “Coping” OR”Empowerment” OR “Telehealth+”) OR “Health Education” OR “Consumer Participation”) OR TI (“self administration*” OR “self care” OR “self efficac*” OR “self manag*” OR selfmanag* OR “self monitor*” OR selfmonitor* OR “self diagnos*” OR selfdiagnos* OR “self assess*” OR selfassess* OR “self direct*” OR selfdirect* OR “self help*” OR empower* OR enablement OR “health education” OR “patient education” OR “patient participation” OR coach* OR “health promot*” OR (((group OR peer) N3 (support* OR advice* OR monitor* OR intervention* OR train* OR instruction* OR consult* OR assist* OR educat* OR information* OR skill*))) OR (((patient OR patients) N3 (centered OR centred OR focus* OR directed OR coach* OR engage* OR involve* OR orient* OR participat*))) OR (((patient OR patients) N3 (educat* OR train* OR instruct* OR teach*))) OR ((management* N3 (plan OR program* OR disease))) OR ((symptom* N3 (management OR directed OR focus*))) OR (((personalized OR personalized) N3 care)) OR telemedicine OR ehealth OR “e health” OR mhealth OR “m health” OR “shared decision*” OR “sharing decision*” OR “informed decision*” OR “informed choice*” OR “decision aid*” OR (((share* OR sharing* OR informed*) N3 (decision* OR deciding* OR choice*))) OR AB (“self administration*” OR “self care” OR “self efficac*” OR “self manag*” OR selfmanag* OR “self monitor*” OR selfmonitor* OR “self diagnos*” OR selfdiagnos* OR “self assess*” OR selfassess* OR “self direct*” OR selfdirect* OR “self help*” OR empower* OR enablement OR “health education” OR “patient education” OR “patient participation” OR coach* OR “health promot*” OR (((group OR peer) N3 (support* OR advice* OR monitor* OR intervention* OR train* OR instruction* OR consult* OR assist* OR educat* OR information* OR skill*))) OR (((patient OR patients) N3 (centered OR centred OR focus* OR directed OR coach* OR engage* OR involve* OR orient* OR participat*))) OR (((patient OR patients) N3 (educat* OR train* OR instruct* OR teach*))) OR ((management* N3 (plan OR program* OR disease))) OR ((symptom* N3 (management OR directed OR focus*))) OR (((personalized OR personalized) N3 care)) OR telemedicine OR ehealth OR “e health” OR mhealth OR “m health” OR “shared decision*” OR “sharing decision*” OR “informed decision*” OR “informed choice*” OR “decision aid*” OR (((share* OR sharing* OR informed*) N3 (decision* OR deciding* OR choice*)))	473,148
S1	(MH “Pulmonary Disease, Chronic Obstructive+”) OR TI (obstructive N3 (lung OR pulmonary OR airway) N3 disease*) OR “acute exacerbation*” OR AECB OR “restrictive lung disease*” OR COAD) OR AB (obstructive N3 (lung OR pulmonary OR airway) N3 disease*) OR “acute exacerbation*” OR AECB OR “restrictive lung disease*” OR COAD)	22,225

**Search History Cochrane Central Trial Register 8 December 2018**

Search	Query	Items found
#4	#3 Limits Publication Year from 2010 to 2018, with Cochrane Library publication date from Jan 2010 to Dec 2018,	1531
#3	#1 AND #2	2379
#2	(self NEXT administration* OR self NEXT care OR self NEXT efficac* OR self NEXT manag* OR selfmanag* OR self NEXT monitor* OR selfmonitor* OR self NEXT diagnos* OR selfdiagnos* OR self NEXT assess* OR selfassess* OR self NEXT direct* OR selfdirect* OR self NEXT help* OR empower* OR enablement OR health NEXT education OR patient NEXT education OR patient NEXT participation OR coach* OR health NEXT promot*):ti,ab,kw OR ((group OR peer) NEAR/3 (support* OR advice* OR monitor* OR intervention* OR train* OR instruction* OR consult* OR assist* OR educat* OR information* OR skill*)):ti,ab,kw OR ((patient OR patients) NEAR/3 (centered OR centred OR focus* OR directed OR coach* OR engage* OR involve* OR orient* OR participat*)):ti,ab,kw OR ((patient OR patients) NEAR/3 (educat* OR train* OR instruct* OR teach*)):ti,ab,kw OR (management* NEAR/3 (plan OR program* OR disease)):ti,ab,kw OR (symptom* NEAR/3 (management OR directed OR focus*)):ti,ab,kw OR ((personalized OR personalized) NEAR/3 care):ti,ab,kw OR (telemedicine OR ehealth OR “e-health” OR mhealth OR “m-health” OR shared NEXT decision* OR sharing NEXT decision* OR informed NEXT decision* OR informed NEXT choice* OR decision NEXT aid*):ti,ab,kw OR ((share* OR sharing* OR informed*) NEAR/3 (decision* OR deciding* OR choice*)):ti,ab,kw OR (self NEXT administration* OR self NEXT care OR self NEXT efficac* OR self NEXT manag* OR selfmanag* OR self NEXT monitor* OR selfmonitor* OR self NEXT diagnos* OR selfdiagnos* OR self NEXT assess* OR selfassess* OR self NEXT direct* OR selfdirect* OR self NEXT help* OR empower* OR enablement OR health NEXT education OR patient NEXT education OR patient NEXT participation OR coach* OR health NEXT promot*):ti,ab,kw OR ((group OR peer) NEAR/3 (support* OR advice* OR monitor* OR intervention* OR train* OR instruction* OR consult* OR assist* OR educat* OR information* OR skill*)):ti,ab,kw OR ((patient OR patients) NEAR/3 (centered OR centred OR focus* OR directed OR coach* OR engage* OR involve* OR orient* OR participat*)):ti,ab,kw OR ((patient OR patients) NEAR/3 (educat* OR train* OR instruct* OR teach*)):ti,ab,kw OR (management* NEAR/3 (plan OR program* OR disease)):ti,ab,kw OR (symptom* NEAR/3 (management OR directed OR focus*)):ti,ab,kw OR ((personalized OR personalized) NEAR/3 care):ti,ab,kw OR (telemedicine OR ehealth OR “e-health” OR mhealth OR “m-health” OR shared NEXT decision* OR sharing NEXT decision* OR informed NEXT decision* OR informed NEXT choice* OR decision NEXT aid*):ti,ab,kw OR ((share* OR sharing* OR informed*) NEAR/3 (decision* OR deciding* OR choice*)):ti,ab,kw	120,972
#1	(obstructive NEAR/3 (lung OR pulmonary OR airway) NEAR/3 disease*) OR acute NEXT exacerbation* OR AECB OR restrictive NEXT lung NEXT disease* OR COAD:ti,ab,kw	13,914

## Appendix B

**Inclusion Criteria**	**Considerations**
1 The article is in an eligible format and language	The article should be written in English or Spanish. Invalid article types such as study proposals or designs as well as dissertations should be excluded.
2 Type of study is a randomized controlled trial.	If at the full-text assessment or data extraction phase you encounter a study labelled as quasi-random, quasi-randomized, or quasi-experimental, please: 1 Before excluding, check whether the study actually meets the methodological criteria for being classified as an RCT (i.e., it is a prospective study, it compares two or more interventions (one or more can be control/no treatment), and there is an allocation sequence that is generated via a random or quasi-random system). Some studies may self-classify as quasi-experimental/random based on not implementing a sample size calculation or for evening out baseline characteristics, e.g., gender or age; none of these are grounds for excluding based on study design. 2 Studies that recruit a convenience sample can be included.
3 Participants are all 18 years or older, or at least 80% is 18 years or older.	
4 Patients should be diagnosed with one of the following: - COPD - Chronic Obstructive Pulmonary Disease - COAD - Chronic Obstructive Airway Disease - Chronic Obstructive Lung Disease - Chronic Airflow Obstruction - (Chronic) Bronchitis - Pulmonary Emphysema - Emphysema	At least 80% of the sample should have one of these diagnoses. In case of mixed populations: If the study population is mixed, outcomes should be reported by disease and not for the mixed population only. Pregnant women are excluded
5 Type of population is patients or caregivers.	
6 It is a self-management intervention Self-management is defined as “what individuals, families and communities do with the intention to promote, maintain, or restore health and to cope with illness and disability with or without the support of health professionals. It includes but is not limited to self-prevention, self-diagnosis, self-medication and self-management of illness and disability...”.). Self-management interventions SMIs are systematically provided by healthcare staff or other patients or lay persons to increase patients’ skills and confidence in managing their chronic condition. SMIs aim to equip patients (and their informal caregivers whenever appropriate) to actively participate in the management of their chronic condition.	
7 The self-management intervention should be the main focus of the intervention.	If the self-management intervention is not the more central component, it is just one of many other components → exclude study. For example, if an intervention is introducing a disease management program, which includes elements of SMI but has stronger component of organizational change, it should be EXCLUDED. If the self-management intervention is compared with other components but outcomes are reported separately, it should be included. For example, if in a case of disease management program the authors independently analyse the effectiveness of the SM components, the study can be included.
8 The RCT includes, at least, one of our outcomes of interest for COPD: - Knowledge - Caregiver knowledge and competence - Health literacy - Self-efficacy - Patient activation - Smoking Cessation - Self-monitoring - Taking medication or other treatment as advised (adherence) and adherence to regular visits (like rehabilitation) - COPD symptoms (short-term) - Sleep quality - Exacerbation - Adverse events - Mortality - Quality of life - Caregiver quality of life (including burden) - Activities of daily living: including sex life, social activities, and work (usual activities) - Physical activity, also including muscle strength - Coping with the disease, including depression and anxiety - Participation and decision-making - Number of all cause hospital admissions - Number of COPD-related hospital admissions - Number of emergency room visits and admissions - Cost effectiveness and resources use	

## Appendix C

**Table A1:** COMPAR-EU SMI taxonomy (Source: Orrego, C. Health Expect. 2021).

Domain 1: Self-Management Intervention Characteristics	
Subdomains	Ingredients
1.1 Support techniques	Sharing information, Skills training, Stress and/or emotional management, Shared decision-making, Goal setting and action planning, Enhancing problem solving skills, Self-monitoring training and feedback, Using prompts and reminders, Encouraging use of services, Providing equipment, Social support, Coaching, and motivational interviewing
1.2 Support delivery methods	Set of five subdomains
1.2.1 Type of encounter	Clinical visit, Support session, Self-guided intervention
1.2.2 Support delivery mode	Face-to-face intervention, Distance/remote intervention, Phone (calls only), Smartphone, Internet, Specific devices
1.2.3 Time of communication	Synchronous–Asynchronous
1.2.4 Recipient	Individual, Group, Specific population
1.2.5 Type of provider	Physician–Nurse–Pharmacist–Physiotherapist–Occupational therapist–Social worker–Psychologist -Dietician/nutritionist–Healthcare Assistant–Peer-Lay Person–Service
1.3 Location	Hospital, Long-term care centre/nursing home, Community-based care, Home, Primary care centre, Outpatient Setting, Workplace
**Domain 2: Expected patient (or caregiver) self-management behaviours**	
2.1 Lifestyle-related behaviours	Eating behaviours, Physical activity/exercise, Smoking cessation or reduction, Cessation or reduction of the consumption of alcohol or other harmful substances, Healthy sleep habits
2.2 Clinical management	Disease-specific behaviours, Self-monitoring, Medication use and adherence, Early recognition of symptoms, Asking for professional help or emergency care when needed, Device Management, Physical management
2.3 Psychological management	Handling/managing emotions
2.4 Social Management	Fitting in at work, Social Roles, Being able to work
2.5 Working with healthcare and/or social care providers	Communicating with healthcare and/or social care providers
**Domain 3: Outcomes to measure the effect of a self-management intervention**	
3.1 Basic empowerment components	Level of knowledge, Level of health literacy, Level of skill acquisition, Level of self-efficacy, Level of patient activation
3.2 Level of adherence to expected self-management behaviours	Lifestyle-related behaviours, Clinical self-management behaviours, Psychological self-management behaviours, Social self-management behaviours, Interactions and communication with healthcare and/or social care providers
3.3 Clinical outcomes	Disease progression (clinical markers, symptoms), Complications, Adverse Events, Mortality
3.4 Patient and informal caregivers’ quality of life	Overall quality of life, Physical functioning, Psychological and emotional role functioning, Social functioning, Sexual functioning, Burden of treatment
3.5 Perceptions of and/or satisfaction with care	Overall satisfaction with self-management intervention, Perception of being well and sufficiently informed (quality of information provision), Perception of patient–provider relationship, Personalized care
3.6 Healthcare use	Type and number of visits, hospital admissions and readmissions, emergency care
3.7 Cost	Healthcare costs for patient, healthcare costs, direct non-medical costs, societal costs
**Domain 4: Target population**	
4.1 As defined by intervention recipient	Patients, informal caregivers, or family caregivers
4.2 As defined by disease-related characteristics	Time since diagnosis, disease severity, comorbidity, and multimorbidity
4.3 As defined by socioeconomic or demographic characteristics	Socioeconomic status, cultural group, health literacy, digital literacy, biological sex or gender, age, living situation

## Appendix D

CORE outcome set for COPD ( Source: Camus, E PLoS ONE 2021).

**Outcome (COS)**	**Definition**
Knowledge	Relates to knowledge about COPD in general and COPD self-management, or the way care for COPD is organized and this both for patients and their social network.
Self-efficacy	A person’s belief that s/he is capable of doing something, often related to a specific goal s/he wants to achieve; feeling of confidence and of being in control.
Patient activation	The knowledge, skills, and confidence a person has on managing their own health and healthcare, including a feeling of being responsible for taking care of their own health.
Lung function	FEV1
Mortality	
Taking medication or other treatment as advised (adherence) and adherence to regular visits	The extent to which a patient follows the prescribed treatment, such as taking medication as advised and following lifestyle advice, and extent of attending scheduled visits.
Smoking cessation	Stopping smoking (and/or smoking less).
COPD symptoms (short-term)	Extent of symptoms relief (in the short-term, including cough; breathlessness, among others).
Physical activity—muscle strength	Referral/participation in a pulmonary rehabilitation program: physical activity, physical activities, muscle strength linked with exercise capacity plus an overall support.
Sleep quality	Sleep quality contains interrupted sleep, sleep problems, sleep quality (as overall), and sleepiness.
Exacerbation	Increased breathlessness, mucus/phlegm/sputum production, and change in color of sputum and feeling out of breath.
Caregiver quality of life (including burden)	Caregiver quality of life and the burden that he/she feels from the caregiver’s tasks.
Activities of daily living: including sex life, social activities, and work (usual activities)	Being able to do usual activities, such as personal hygiene, housework, sex, managing finances, social activities, and work.
Coping with the disease, including depression and anxiety	How well a person feels able to cope/manage with stress or other difficulties caused by the disease, including depression and anxiety.
Number of emergency room visits and admissions	Number of visits to emergency department visits and hospital admissions.
